# Improving inpatient postpartum depression screening: results from a quality improvement initiative

**DOI:** 10.1007/s00737-025-01591-0

**Published:** 2025-05-15

**Authors:** Rebecca Woofter, Gwendolyn Hill, Emily Wong, Tiffani J. Bright, Catherine Bresee, Sarah J. Kilpatrick, Eynav E. Accortt

**Affiliations:** 1 Department of Community Health Sciences, Fielding School of Public Health, University of California Los Angeles, Los Angeles, CA USA; 2 Reproductive Psychology Program, Department of Obstetrics and Gynecology, Cedars Sinai Medical Center, Los Angeles, CA USA; 3 Department of Computational Biomedicine, Center for Artificial Intelligence Research, Cedars Sinai Medical Center, Los Angeles, CA USA; 4 Biostatistics Shared Resources, Cedars-Sinai Medical Center, Los Angeles, CA USA

**Keywords:** Postpartum depression, Postpartum anxiety, Mental health screening, Edinburgh postnatal depression scale, Patient health questionnaire

## Abstract

**Purpose:**

Screening is necessary to identify patients with postpartum depression or anxiety and facilitate access to mental healthcare. This study evaluated a quality improvement initiative for inpatient postpartum depression screening, which was implemented to better identify patients in need of mental healthcare. This initiative replaced the Patient Health Questionnaire (PHQ-9) administered verbally by nurses with the Edinburgh Postnatal Depression Scale (EPDS-10) self-administered by patients via iPads.

**Methods:**

Electronic medical records for patients who delivered June 2020-October 2023 at Cedars-Sinai were analyzed (*N* = 17,403). Differences in completed screenings, positive depression screenings, suicidal ideation, and social work referrals before and after the initiative were examined with chi-square tests and multiple logistic regression models. Factors associated with positive anxiety screenings on the EPDS-10 were also identified.

**Results:**

Overall, 98.6% of patients completed depression screenings. Among those who completed screenings, positive depression screenings increased from 4.0 to 11.4%, identified suicidal ideation increased from 0.2 to 1.1%, and social work referrals increased from 64.1 to 84.0%, before and after the initiative. Post-initiative, patients had higher odds of positive depression screenings, indicating suicidal ideation, and receiving social work referrals compared to pre-initiative. Among those who completed EPDS-10 screenings, 31% were positive for anxiety.

**Conclusions:**

Compared to the PHQ-9 administered verbally by nurses, the EPDS-10 self-administered by patients via iPads resulted in higher rates of positive depression screenings, identified suicidal ideation, and social work referrals. It is imperative to accurately screen patients for postpartum depression and anxiety to facilitate access to mental healthcare and address morbidity and mortality.

**Clinical trial number:**

Not applicable.

## Highlights

Cedars-Sinai replaced inpatient screening verbally with the PHQ-9 with the EPDS-10 self-administered by patients via iPads.

Both before and after the quality improvement initiative, about 99% of patients completed mental health screenings.

Post-initiative, positive depression screenings, identified suicidal ideation, and social work referrals increased compared to pre-initiative.

## Introduction

Postpartum depression (PPD) affects up to 25% of women and other birthing people in the U.S. (Al-Abri et al. 2023; Bauman et al. 2020; Sahebi et al. 2024). Though less often studied, postpartum anxiety (PPA) also affects up to 20% of patients (Wouk et al. 2017; Dennis et al. 2017), and many patients experience comorbid PPD/PPA (Falah-Hassani et al. 2017).

Without treatment, PPD/PPA can result in adverse maternal and infant outcomes (Slomian et al. 2019; Accortt et al. 2024). Approximately one-quarter of all pregnancy-related deaths in the U.S. are attributed to mental health conditions (Trost et al. 2022). Although treatment can mitigate the consequences of PPD/PPA, less than half of those with PPD/PPA receive mental health treatment (Cox et al. 2016; Declercq et al. 2022; Haight et al. 2024). Despite facing higher rates of PPD/PPA, Black patients are less likely than their White counterparts to receive mental health treatment (Declercq et al. 2022; Haight et al. 2024).

Screening for PPD/PPA is a vital step in facilitating access to mental health treatment. Indeed, the American College of Obstetricians and Gynecologists recommends mental health screening during pregnancy and postpartum (Committee on Obstetric Practice 2018). However, approximately one-third of postpartum patients are not screened for mental health (Sidebottom et al. 2021). In 2019, California joined several other states in mandating perinatal mental health screening (Griffen et al. 2021).

Screening for PPD is primarily conducted using either the Patient Health Questionnaire (PHQ-9) or the Edinburgh Postnatal Depression Scale (EPDS-10) (Committee on Obstetric Practice 2018). Both instruments measure symptoms of depression and suicidal ideation. Additionally, the anxiety subscale within the EPDS-10, also known as the EPDS-3, captures anxiety symptoms, allowing healthcare providers to identify both depression and anxiety using one instrument (Smith-Nielsen et al. 2021).

Cedars-Sinai is a nonprofit hospital in California serving approximately 6,500 deliveries annually. Beginning in 2017, Cedars-Sinai implemented the Postpartum Depression Screening, Education, and Referral Quality Improvement Program to improve mental health screening and referral practices in the postpartum unit (Accortt et al. 2022). Although nearly all patients completed PPD screening after this program, less than 5% of patients screened positive for depression, which is much lower than national rates. As part of the ongoing program, in February 2022, Cedars-Sinai began an additional quality improvement (QI) initiative replacing the PHQ-9 conducted verbally by nurses with the EPDS-10 completed independently by patients on iPads. The EPDS-10 is available on iPads in English and on paper in other languages. The change in screening instrument was intended to increase the accuracy of screening, as the EPDS-10 was specifically developed for perinatal populations (Cox et al. 1987). The change in screening modality was intended to reduce the burden on nurses and increase patient privacy, thereby encouraging both screening completion and honest responses (Hsieh et al. 2021; Clarke et al. 2024; Guille et al. 2024).

This study aimed to examine the outcomes of this postpartum inpatient screening QI initiative. Specifically, this study assessed differences before and after the initiative as well as sociodemographic and health characteristics associated with (1) screening completion, (2) positive depression screenings, (3) suicidal ideation, and (4) social work referrals. This study also identified sociodemographic and health characteristics associated with (5) positive anxiety screening.

## Methods

### Dataset

This analysis leveraged electronic medical records (EMR) data from patients over 18 y old who delivered at Cedars-Sinai between June 1, 2020 and October 31, 2023. This period was selected to include 20 months of data both before and after the initiative. Patients who experienced fetal demise were excluded, as they receive automatic social work referrals and do not complete the typical mental health screening process, for a total of 18,039 patients. Patients missing data on any study variables were also excluded, resulting in an analytic population of 17,403 patients. This study was deemed exempt by the Institutional Review Board at Cedars-Sinai.

### Measures

The outcomes of this study included screening completion, positive depression screenings, suicidal ideation, social work referrals, and positive anxiety screenings. All outcomes except social work referrals were calculated based on responses to the screening instrument. *Screening completion* was coded as patients completing all of the screening questions versus not.

Among those who completed screenings, scores were categorized based on the clinical cutoffs used at Cedars-Sinai. Notably, these cutoffs are lower than research-based cutoffs. These lower cutoffs were intentionally selected by clinicians at Cedars-Sinai to ensure that all patients with possible PPD/PPA are identified and referred to social work (Accortt et al. 2022). Scores on the PHQ-9 range from zero to 27 and were categorized as low risk (0–4), moderate risk (5–12), or high risk (13–27 or any suicidal ideation) of PPD. Scores on the EPDS-10 range from zero to 30 and were categorized as low risk (0–7), moderate risk (8–12), or high risk (13–30 or any suicidal ideation) of PPD. *Positive depression screening* was coded as moderate or high risk of PPD versus low risk.

The final item on both the PHQ-9 and the EPDS-10 measure suicidal ideation, with any responses above zero on this item indicating suicidal ideation. *Suicidal ideation* was coded as scores of at least one versus zero on the suicidal ideation question.

At Cedars-Sinai, approximately 50% of perinatal patients are offered social work referrals for a variety of reasons, including PPD/PPA risk, domestic violence, substance use, pregnancy loss, and admission to the Maternal Fetal Care Unit (MFCU) and the Neonatal Intensive Care Unit (NICU). Any patients whose screening indicates a high risk of PPD are automatically referred to social work. Patients in the low and moderate PPD risk categories are offered optional social work referrals. *Social work referrals* were coded as receiving social work referrals versus not.

Among those who completed the EPDS-10 screening in the post-initiative period, the EPDS-3 anxiety subscale score was calculated. Scores on the EPDS-3 range from zero to nine. *Positive anxiety screening* was coded as EPDS-3 scores of at least three versus less than three.

Sociodemographic and health characteristics shown to be associated with PPD/PPA were included as covariates in multivariable analyses (Hutchens and Kearney 2020; Bauman et al. 2020; Haight et al. 2024). Sociodemographic characteristics included age (18–25, 26–30, 31–35, 36–40, 41+), race/ethnicity (Asian, Black, Hispanic, Multiracial/Other Race, White), relationship status (partnered versus single), and insurance coverage (private versus public). Health characteristics included parity (nulliparous versus multiparous), history of depression/anxiety (yes versus no), pregnancy complications (gestational hypertension, preeclampsia/eclampsia, and/or gestational diabetes versus none), delivery type (vaginal versus cesarean), low birthweight (LBW; birthweight < 2500 versus > = 2500 g), preterm birth (PTB; birth before 37 weeks of gestation versus after), and admission to the MFCU (yes versus no) and NICU (yes versus no).

### Analyses

Descriptive frequencies and chi-square tests were conducted to characterize the patient population and examine differences in outcomes across independent variables. To address the study aims, multivariable logistic regression models were conducted for each outcome, adjusting for all sociodemographic and health characteristics listed above. Analysis were performed in STATA 16/SE.

## Results

Table 1 depicts the sociodemographic and health characteristics in the patient population. In both the pre-initiative and post-initiative time periods, most patients were between 31 and 40 years old, White, privately insured, partnered, and nulliparous. About 13% of patients had histories of depression/anxiety and about one-fifth had pregnancy complications. Around one-third of patients had cesarean deliveries. About 6% of infants had LBW and about 7% had PTB. Approximately 10% of patients were admitted to the MFCU and around 14% of infants were admitted to the NICU. Post-initiative, statistically significantly more patients were above age 35, non-White, single, nulliparous, and had pregnancy complications, cesarean deliveries, LBW infants, PTB infants, MFCU admissions, and NICU admissions, compared to pre-initiative.

**Table 1:** Patient characteristics before and after the quality improvement initiative (Patients Delivering at Cedars-Sinai, June 2020–October 2023)

	Total (*N* = 17,403)	Pre-Initiative (*N* = 9,534)	Post-Initiative (*N* = 7,869)	*P*-Value
	*N* (%)			
**Maternal Age**				< 0.001
* 18–25*	845 (4.9)	486 (5.1)	359 (4.6)	
* 26–30*	2561 (14.7)	1400 (14.7)	1161 (14.8)	
* 31–35*	7168 (41.2)	4026 (42.2)	3142 (39.9)	
* 36–40*	5415 (31.1)	2914 (30.6)	2501 (31.8)	
* 41+*	1414 (8.1)	708 (7.4)	706 (9.0)	
**Maternal Race/Ethnicity**				0.001
* Asian*	2107 (12.1)	1144 (12.0)	963 (12.2)	
* Black*	1218 (7.0)	659 (6.9)	559 (7.1)	
* Hispanic*	2792 (16.0)	1457 (15.3)	1335 (17.0)	
* Multiracial/Other*	1414 (8.1)	734 (7.7)	680 (8.6)	
* White*	9872 (56.7)	5540 (58.1)	4332 (55.1)	
**Insurance Coverage**				0.797
* Private Insurance*	16,872 (97.0)	9246 (97.0)	7626 (96.9)	
* Public Insurance*	531 (3.1)	288 (3.0)	243 (3.1)	
**Relationship Status**				< 0.001
* In a Relationship*	15,586 (89.6)	8636 (90.6)	6950 (88.3)	
* Single*	1817 (10.4)	898 (9.4)	919 (11.7)	
**Parity**				< 0.001
* Multiparous*	7114 (40.9)	4333 (45.5)	2781 (35.3)	
* Nulliparous*	10,289 (59.1)	5201 (54.6)	5088 (64.7)	
**History of Depression/Anxiety**				0.150
* No*	15,103 (86.8)	8242 (86.5)	6861 (87.2)	
* Yes*	2300 (13.2)	1292 (13.6)	1008 (12.8)	
**Pregnancy Complications**				< 0.001
* No*	13,580 (78.0)	7592 (79.6)	5988 (76.1)	
* Yes*	3823 (22.0)	1942 (20.4)	1881 (23.9)	
**Delivery Type**				< 0.001
* Vaginal Delivery*	11,778 (67.7)	6688 (69.9)	5110 (64.9)	
* Cesarean Delivery*	5625 (32.3)	2866 (30.1)	2759 (35.1)	
**Birthweight**				0.001
* Normal Birthweight*	16,365 (94.0)	9019 (94.6)	7346 (93.4)	
* Low Birthweight*	1038 (6.0)	515 (5.4)	523 (6.7)	
**Gestational Age**				0.003
* Term Birth*	16,140 (92.7)	8892 (93.3)	7248 (92.1)	
* Preterm Birth*	1263 (7.3)	642 (6.7)	621 (7.9)	
**MCFU Admission**				< 0.001
* Not Admitted*	15,720 (90.3)	8742 (91.7)	6978 (88.7)	
* Admitted*	1683 (9.7)	792 (8.3)	891 (11.3)	
**NICU Admission**				< 0.001
* Not Admitted*	14,913 (85.7)	8252 (86.6)	6661 (84.7)	
* Admitted*	2490 (14.3)	1282 (13.5)	1208 (15.4)	
**Screening Completion**				0.528
* Incomplete*	252 (1.5)	143 (1.5)	109 (1.4)	
* Complete*	17,151 (98.6)	9391 (98.5)	7760 (98.6)	
**Social Work Referral**				< 0.001
* Not Referred*	4680 (26.9)	3422 (35.9)	1258 (16.0)	
* Referred*	12,723 (73.1)	6112 (64.1)	6611 (84.0)	
**Among Those Who Completed Screenings**				
**Depression Screening Result**				< 0.001
* Negative*	15,899 (92.7)	9020 (96.1)	6879 (88.7)	
* Positive*	1252 (7.3)	371 (4.0)	881 (11.4)	
**Depression Screen Risk**				< 0.001
* Low*	15,899 (92.7)	9020 (96.1)	6879 (88.7)	
* Moderate*	986 (5.8)	326 (3.5)	660 (8.5)	
* High*	266 (1.6)	45 (0.5)	221 (2.9)	
**Anxiety Screening Result**				-
* Negative*	-	-	5381 (69.3)	
* Positive*	-	-	2379 (30.7)	
**Suicidal Ideation**				< 0.001
* Not Endorsed*	17,046 (99.4)	9370 (99.8)	7676 (98.9)	
* Endorsed*	105 (0.6)	21 (0.2)	84 (1.1)	

Table 1 also depicts the frequency of each outcome with p-values from chi-square tests. Overall, 98.6% of patients completed screenings (98.5% pre-initiative vs. 98.6% post-initiative, *p* = 0.528). Among patients who completed screenings, 7.3% were positive for depression (4.0% pre-initiative vs. 11.4% post-initiative, *p* < 0.001) and 0.6% indicated suicidal ideation (0.2% pre-initiative vs. 1.1% post-initiative, *p* < 0.001). Additionally, 73.1% of patients received social work referrals (63.6% pre-initiative vs. 84.1% post-initiative, *p* < 0.001). Among those who completed screenings in the post-initiative period, 30.7% were positive for anxiety.

Table 2 compares sociodemographic and health characteristics across screening completion status during the entire study period. Several factors were statistically significantly associated with completed screenings: race/ethnicity, insurance coverage, relationship status, parity, pregnancy complications, LBW infants, PTB infants, MFCU admissions, and NICU admissions. Notably, a larger proportion of those who did not complete screenings were Black (11.5% vs. 6.9%) and Hispanic (17.5% vs. 16.0%) compared to those who did complete screenings.

**Table 2:** Sociodemographic and health characteristics of patients who completed and did not complete a screening (Patients Delivering at Cedars-Sinai, June 2020–October 2023; *N* = 17,403)

	Did Not Complete Screening	Completed Screening	*P*-Value
	*N* (%)		
**Maternal Age **			0.178
* 18–25*	9 (3.6)	836 (4.9)	
* 26–30*	50 (19.8)	2511 (14.6)	
* 31–35*	99 (39.3)	7069 (41.2)	
* 36–40*	72 (28.6)	5343 (31.2)	
* 41+*	22 (8.7)	1392 (8.1)	
**Maternal Race/Ethnicity**			0.030
* Asian*	24 (9.5)	2083 (12.2)	
* Black*	29 (11.5)	1189 (6.9)	
* Hispanic*	44 (17.5)	2748 (16.0)	
* Multiracial/Other*	15 (6.0)	1399 (8.2)	
* White*	140 (55.6)	9732 (56.7)	
**Insurance Coverage**			0.002
* Private Insurance*	236 (93.7)	16,636 (97.0)	
* Public Insurance*	16 (6.4)	515 (3.0)	
**Relationship Status**			< 0.001
* In a Relationship*	208 (82.5)	15,378 (89.7)	
* Single*	44 (17.5)	1773 (10.3)	
**Parity**			0.003
* Multiparous*	126 (50.0)	6988 (40.7)	
* Nulliparous*	126 (50.0)	10,163 (59.3)	
**History of Depression/Anxiety**			0.237
* No*	225 (89.3)	14,878 (86.8)	
* Yes*	27 (10.7)	2273 (13.3)	
**Pregnancy Complications**			< 0.001
* No*	166 (65.9)	13,414 (78.2)	
* Yes*	86 (34.1)	3737 (21.8)	
**Delivery Type**			0.050
* Vaginal Delivery*	185 (73.4)	11,593 (67.6)	
* Cesarean Delivery*	67 (26.6)	5558 (32.4)	
**Birthweight**			< 0.001
* Normal Birthweight*	198 (78.6)	16,167 (94.3)	
* Low Birthweight*	54 (21.4)	984 (5.7)	
**Gestational Age**			< 0.001
* Term Birth*	187 (74.2)	15,953 (93.0)	
* Preterm Birth*	65 (25.8)	1198 (7.0)	
**MCFU Admission**			< 0.001
* Not Admitted*	139 (55.2)	15,581 (90.9)	
* Admitted*	113 (44.8)	1570 (9.2)	
**NICU Admission**			< 0.001
* Not Admitted*	191 (75.8)	14,722 (85.8)	
* Admitted*	61 (24.2)	2429 (14.2)	

Table 3 depicts the odds of positive depression screenings among those who completed screenings, adjusting for sociodemographic and health covariates. The odds of positive depression screenings post-initiative were 3.03 times the odds pre-initiative (*p* < 0.001). Additionally, Asian, Black, and Hispanic patients had statistically significantly higher odds of positive depression screenings than White patients. Several other factors were also statistically significantly associated with higher odds of positive depression screenings: being single, histories of depression/anxiety, cesarean deliveries, and NICU admissions.

**Table 3:** Multivariable logistic regression estimating odds of positive depression screening among those who completed screenings before and after the initiative (Patients Delivering at Cedars-Sinai, June 2020–October 2023; *N* = 17,151)

	Adjusted Odds Ratio (95% Confidence Interval)	*P*-Value
**Time Period**		
* Pre-Initiative*	Ref	Ref
* Post-Initiative*	3.03 (2.67, 3.44)	< 0.001
**Maternal Age**		
* 18–25*	Ref	Ref
* 26–30*	1.17 (0.84, 1.64)	0.341
* 31–35*	1.34 (0.98, 1.83)	0.064
* 36–40*	1.27 (0.93, 1.75)	0.137
* 41+*	1.29 (0.90, 1.85)	0.161
**Maternal Race/Ethnicity**		
* Asian*	1.74 (1.47, 2.05)	< 0.001
* Black*	1.30 (1.03, 1.65)	0.025
* Hispanic*	1.30 (1.10, 1.54)	0.002
* Multiracial/Other/Unknown*	1.12 (0.89, 1.39)	0.336
* White*	Ref	Ref
**Insurance Coverage**		
* Private Insurance*	Ref	Ref
* Public Insurance*	1.22 (0.89, 1.68)	0.210
**Relationship Status**		
* In a Relationship*	Ref	Ref
* Single*	1.35 (1.12, 1.62)	0.001
**Parity**		
* Multiparous*	Ref	Ref
* Nulliparous*	1.08 (0.95, 1.22)	0.251
**History of Anxiety/Depression**		
* No*	Ref	Ref
* Yes*	1.64 (1.41, 1.92)	< 0.001
**Pregnancy Complications**		
* No*	Ref	Ref
* Yes*	1.04 (0.90, 1.20)	0.612
**Delivery Type**		
* Vaginal Delivery*	Ref	Ref
* Cesarean Delivery*	1.26 (1.11, 1.43)	< 0.001
**Birthweight**		
* Normal Birthweight*	Ref	Ref
* Low Birthweight*	1.10 (0.84, 1.44)	0.490
**Gestational Age**		
* Term Birth*	Ref	Ref
* Preterm Birth*	1.11 (0.85, 1.44)	0.444
**MFCU Admission**		
* Not Admitted*	Ref	Ref
* Admitted*	0.92 (0.74, 1.14)	0.445
**NICU Admission**		
* Not Admitted*	Ref	Ref
* Admitted*	1.32 (1.11, 1.57)	0.001

Table 4 depicts the odds of indicating suicidal ideation among those who completed screenings, adjusting for sociodemographic and health covariates. The odds of indicating suicidal ideation post-initiative were 4.69 times the odds pre-initiative (*p* < 0.001). Additionally, Asian patients had statistically significantly higher odds of indicating suicidal ideation compared to White patients. Several other factors were also statistically significantly associated with higher odds of indicating suicidal ideation: being single, histories of depression/anxiety, and cesarean deliveries.

**Table 4:** Multivariable logistic regression estimating odds of endorsing suicidal ideation among those who completed screenings before and after the initiative (Patients Delivering at Cedars-Sinai, June 2020–October 2023; *N* = 17,151)

	Adjusted Odds Ratio (95% Confidence Interval)	*P*-Value
**Time Period**		
* Pre-Initiative*	Ref	Ref
* Post-Initiative*	4.69 (2.89, 7.59)	< 0.001
**Maternal Age**		
* 18–25*	Ref	Ref
* 26–30*	2.15 (0.62, 7.50)	0.228
* 31–35*	1.94 (0.58, 6.54)	0.284
* 36–40*	1.89 (0.55, 6.45)	0.313
* 41+*	2.14 (0.57, 7.98)	0.258
**Maternal Race/Ethnicity**		
* Asian*	3.12 (1.89, 5.15)	< 0.001
* Black*	1.15 (0.51, 2.57)	0.736
* Hispanic*	1.39 (0.79, 2.45)	0.256
* Multiracial/Other/Unknown*	1.54 (0.76, 3.09)	0.229
* White*	Ref	Ref
**Insurance Coverage**		
* Private Insurance*	Ref	Ref
* Public Insurance*	2.00 (0.88, 4.53)	0.096
**Relationship Status**		
* In a Relationship*	Ref	Ref
* Single*	2.06 (1.22, 3.50)	0.007
**Parity**		
* Multiparous*	Ref	Ref
* Nulliparous*	0.97 (0.64, 1.46)	0.878
**History of Depression/Anxiety**		
* No*	Ref	Ref
* Yes*	2.02 (1.25, 3.24)	0.004
**Pregnancy Complications**		
* No*	Ref	Ref
* Yes*	0.79 (0.49, 1.29)	0.353
**Delivery Type**		
* Vaginal Delivery*	Ref	Ref
* Cesarean Delivery*	1.53 (1.02, 2.30)	0.039
**Birthweight**		
* Normal Birthweight*	Ref	Ref
* Low Birthweight*	1.77 (0.81, 3.85)	0.149
**Gestational Age**		
* Term Birth*	Ref	Ref
* Preterm Birth*	1.32 (0.60, 2.90)	0.490
**MFCU Admission**		
* Not Admitted*	Ref	Ref
* Admitted*	0.94 (0.48, 1.83)	0.857
**NICU Admission**		
* Not Admitted*	Ref	Ref
* Admitted*	1.00 (0.55, 1.80)	> 0.999

**Fig. 1:**
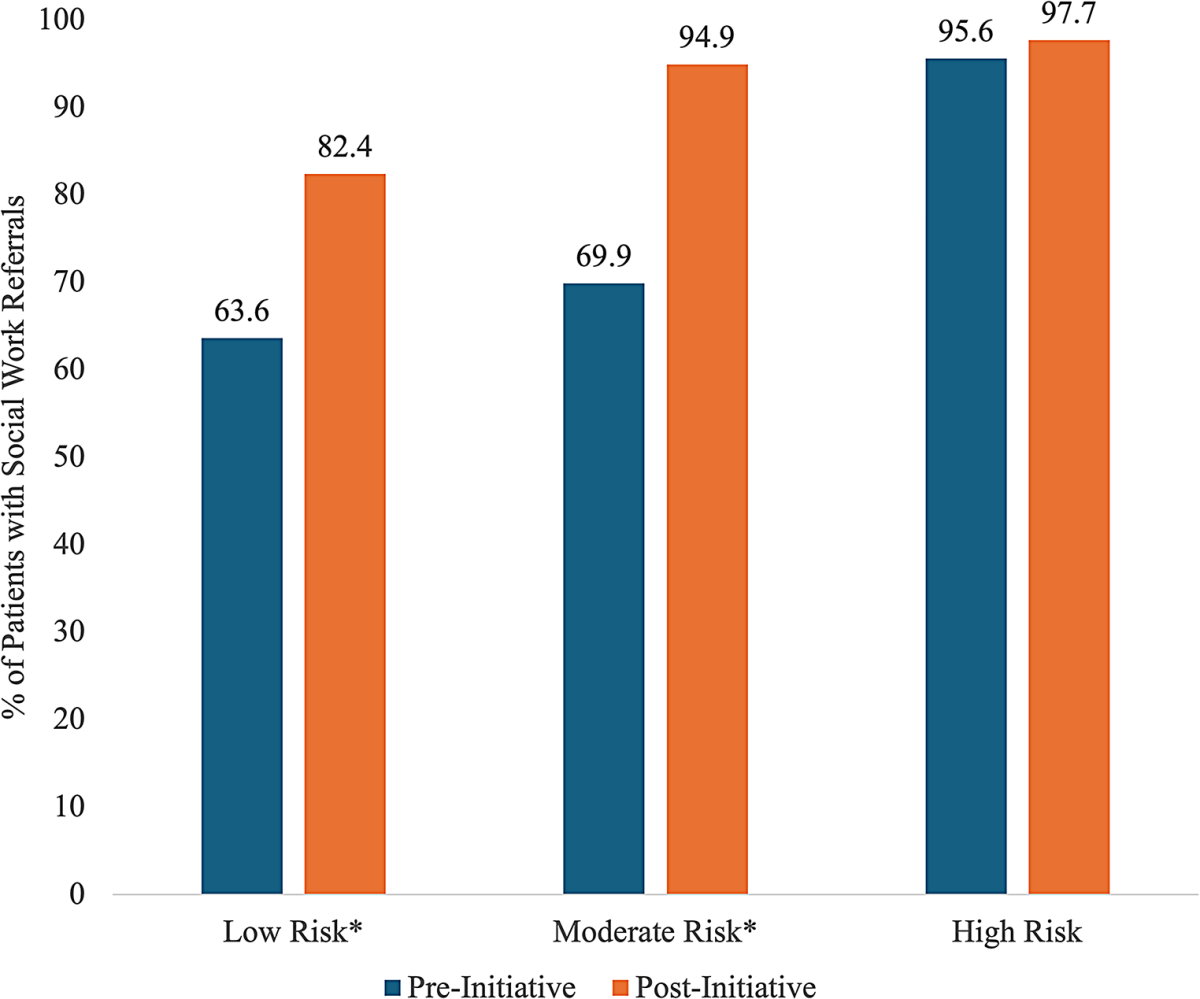
Rates of Social Work Referrals by Depression Score Risk Before and After the Initiative (Patients Delivering at Cedars-Sinai, June 2020–October 2023; *N* = 17,403). *Indicates statistically significant difference (*p* < 0.001)

Figure 1 in the Appendix depicts rates of social work referrals by PPD risk level both pre- and post-initiative. Pre-initiative, 63.6% of low risk patients received social work referrals, along with 69.9% of moderate risk patients and 95.6% of high risk patients. Post-initiative, 82.4% of low risk patients received social work referrals, along with 94.9% of moderate risk patients, and 97.7% of high risk patients. Table 5 depicts the odds of receiving social work referrals, adjusting for sociodemographic and health covariates and positive depression screenings. The odds of receiving social work referrals post-initiative were 2.71 times the odds pre-initiative (*p* < 0.001). Several other factors were also statistically significantly associated with higher odds of social work referrals: positive depression screening, ages 31–40 (compared to 18-25), nulliparity, pregnancy complications, cesarean deliveries, LBW infants, MCFU admissions, and NICU admissions.

**Table 5:** Multivariable logistic regression model estimating odds of social work referral before and after the initiative (Patients Delivering at Cedars-Sinai, June 2020–October 2023; *N* = 17,403)

	Adjusted Odds Ratio (95% Confidence Interval)	*P*-Value
**Time Period**		
* Pre-Initiative*	Ref	Ref
* Post-Initiative*	2.71 (2.51, 2.92)	< 0.001
**Depression Screening Result**		
* Negative*	Ref	Ref
* Positive*	2.35 (1.95, 2.83)	< 0.001
**Maternal Age**		
* 18–25*	Ref	Ref
* 26–30*	0.83 (0.69, 1.01)	0.067
* 31–35*	0.83 (0.69, 1.00)	0.049
* 36–40*	0.82 (0.68, 0.99)	0.038
* 41+*	0.86 (0.69, 1.08)	0.196
**Maternal Race/Ethnicity**		
* Asian*	0.95 (0.85, 1.06)	0.377
* Black*	0.95 (0.82, 1.11)	0.533
* Hispanic*	1.00 (0.90, 1.12)	0.950
* Multiracial/Other/Unknown*	1.00 (0.87, 1.14)	0.952
* White*	Ref	Ref
**Insurance Coverage**		
* Private Insurance*	Ref	Ref
* Public Insurance*	1.11 (0.89, 1.39)	0.367
**Relationship Status**		
* In a Relationship*	Ref	Ref
* Single*	1.11 (0.97, 1.26)	0.133
**Parity**		
* Multiparous*	Ref	Ref
* Nulliparous*	1.35 (1.25, 1.45)	< 0.001
**History of Depression/Anxiety**		
* No*	Ref	Ref
* Yes*	0.96 (0.87, 1.07)	0.495
**Pregnancy Complications**		
* No*	Ref	Ref
* Yes*	1.21 (1.10, 1.34)	< 0.001
**Delivery Type**		
* Vaginal Delivery*	Ref	Ref
* Cesarean Delivery*	1.22 (1.12, 1.32)	< 0.001
**Birthweight**		
* Normal Birthweight*	Ref	Ref
* Low Birthweight*	1.32 (1.05, 1.66)	0.019
**Gestational Age**		
* Term Birth*	Ref	Ref
* Preterm Birth*	1.05 (0.85, 1.29)	0.655
**MFCU Admission**		
* Not Admitted*	Ref	Ref
* Admitted*	3.13 (2.57, 3.80)	< 0.001
**NICU Admission**		
* Not Admitted*	Ref	Ref
* Admitted*	3.08 (2.64, 3.58)	< 0.001

Table 6 depicts the odds of positive anxiety screenings among those who completed the EPDS-10 based on sociodemographic and health covariates. Asian patients had statistically significantly higher odds of positive anxiety screenings than White patients. Several other factors were also statistically significantly associated with higher odds of positive anxiety screenings: ages 31 and above (compared to 18-25), nulliparity, histories of depression/anxiety, cesarean deliveries, PTB infants, and NICU admissions. Additionally, those with MFCU admissions had statistically significantly lower odds of positive anxiety screenings than those without MFCU admissions.

**Table 6:** Within post-initiative group, multivariable logistic regression model estimating odds of positive anxiety screen among those who completed screening (Patients Delivering at Cedars-Sinai, February 2022–October 2023; *N* = 7,760)

	Adjusted Odds Ratio (95% Confidence Interval)	*P*-Value
**Maternal Age**		
* 18–25*	Ref	Ref
* 26–30*	1.25 (0.94, 1.67)	0.126
* 31–35*	1.44 (1.10, 1.89)	0.008
* 36–40*	1.46 (1.11, 1.92)	0.007
* 41+*	1.38 (1.01, 1.88)	0.042
**Maternal Race/Ethnicity**		
* Asian*	1.44 (1.24, 1.67)	< 0.001
* Black*	0.89 (0.72, 1.10)	0.300
* Hispanic*	0.96 (0.84, 1.11)	0.624
* Multiracial/Other/Unknown*	1.06 (0.89, 1.27)	0.495
* White*	Ref	Ref
**Insurance Coverage**		
* Private Insurance*	Ref	Ref
* Public Insurance*	0.76 (0.55, 1.05)	0.091
**Relationship Status**		
* In a Relationship*	Ref	Ref
* Single*	0.99 (0.84, 1.17)	0.932
**Parity**		
* Multiparous*	Ref	Ref
* Nulliparous*	1.40 (1.26, 1.56)	< 0.001
**History of Depression/Anxiety**		
* No*	Ref	Ref
* Yes*	1.56 (1.35, 1.79)	< 0.001
**Pregnancy Complications**		
* No*	Ref	Ref
* Yes*	1.12 (1.00, 1.27)	0.058
**Delivery Type**		
* Vaginal Delivery*	Ref	Ref
* Cesarean Delivery*	1.24 (1.12, 1.38)	< 0.001
**Birthweight**		
* Normal Birthweight*	Ref	Ref
* Low Birthweight*	0.86 (0.67, 1.10)	0.224
**Gestational Age**		
* Term Birth*	Ref	Ref
* Preterm Birth*	1.28 (1.02, 1.61)	0.037
**MFCU Admission**		
* Not Admitted*	Ref	Ref
* Admitted*	0.66 (0.54, 0.79)	< 0.001
**NICU Admission**		
* Not Admitted*	Ref	Ref
* Admitted*	1.21 (1.04, 1.41)	0.012

## Discussion

This study evaluated a QI initiative for postpartum inpatient screening replacing the PHQ-9 administered verbally by nurses with the EPDS-10 self-administered by patients via iPads. As hypothesized, this initiative resulted in increased rates and odds of positive depression screenings, identified suicidal ideation, and social work referrals, although it did not result in increased rates or odds of screening completion.

The rate of screening completion did not differ between pre-initiative and post-initiative. However, even pre-initiative, 98.5% of patients completed screenings. This high rate of completed screenings is due largely to the prior QI program at Cedars-Sinai (Accortt et al. 2022). However, a notably lower proportion of those who completed screenings were Black compared to those who did not complete screenings. Prior literature has shown that Black parents may be reluctant to complete mental health screenings based on fear of involvement of Child Protective Services (CPS), as Black children are more frequently referred for CPS involvement than White children (Putnam-Hornstein et al. 2013; Hsieh et al. 2021). Given their higher risk of perinatal depression and anxiety (Bauman et al. 2020; Haight et al. 2024), it is pivotal to identify appropriate ways to screen Black patients for PPD/PPA. Technology-based perinatal mental health screening may increase comfort for Black patients (Witcraft et al. 2024). Future research should identify best practices for screening people of color, particularly Black patients, including the use of culturally-appropriate and person-centered screening instruments.

Post-initiative, patients had three times the odds of positive depression screenings and nearly five times the odds of indicating suicidal ideation compared to pre-initiative. These findings are consistent with the hypothesized impact of the initiative, given the enhanced privacy of the self-administered screening. Notably, this study used lower positive depression screening score cutoffs than those often used in research (Kroenke et al. 2001; Levis et al. 2020), and thus, the proportion of patients with positive depression screenings in this study includes those with scores that researchers may not consider to indicate true PPD. Still, it is important to use these lower cutoffs in this study evaluating the QI initiative, as these are the cutoffs used for clinical intervention at Cedars-Sinai to identify all patients with potential PPD/PPA (Accortt et al. 2022). Even still, the rate of positive depression screenings in this setting is still below that found in prior literature (Bauman et al. 2020). The lower rate of positive depression screenings at Cedars-Sinai may be due to the patient population, which is primarily White, aged 30–40 years, and privately insured, which are all protective factors for PPD/PPA (Bauman et al. 2020; Haight et al. 2024). It may also be due to the timing of the screening, as this screening is conducted in the hospital after delivery, while most other studies examine screenings conducted later in the postpartum period (Bauman et al. 2020; Haight et al. 2024). Given that patients who deliver at Cedars-Sinai may receive postpartum care in other health systems, patients complete inpatient screening to identify potential concerns for PPD/PPA and make appropriate referrals before hospital discharge.

Post-initiative, patients had nearly three times the odds of receiving social work referrals compared to pre-initiative. This increase is likely due both to the increased rate of positive depression screenings and increased rates of NICU and MFCU admission in the post-initiative period, which all result in automatic social work referrals. It is worth noting that while Cedars-Sinai intentionally refers a large proportion of patients to social work based on concerns related to PPD/PPA, domestic violence, substance use, pregnancy loss, and MFCU and NICU admission, this rate of social work referrals may not be feasible in all healthcare settings. This study is unable to determine whether patients actually received mental healthcare following social work referrals, though this is an important aspect of addressing perinatal mental health and should be explored in future research.

Finally, this study examined sociodemographic and health factors associated with positive anxiety screening. Patients who were older, Asian (versus White), nulliparous, had histories of depression/anxiety, cesarean deliveries, PTB infants, and NICU admissions had higher odds of positive anxiety screening compared to their counterparts. Notably, patients with MFCU admissions had lower odds of positive anxiety screening than those without admissions. This could be due to patients’ acute health needs in the MFCU, and the EPDS-3 not effectively capturing the extent of patients’ distress. This finding warrants further research.

### Limitations & strengths

This analysis has several limitations. First, it is possible that the differences in outcomes pre- and post-initiative were due to external trends rather than the QI initiative. In particular, this study examines deliveries that occurred during the ongoing COVID-19 pandemic, a period marked by heightened stress, impacted care systems, and complications from COVID-19 infection — all of which may increase rates of PPD/PPA (Perzow et al. 2021; Masters et al. 2021; Sahebi et al. 2024). However, given that the early period of the COVID-19 pandemic occurred pre-initiative, if PPD/PPA rates were elevated at this time, the findings presented here may underestimate the true impact of the initiative. Second, the EMR data in this study aggregate racial groups which may have different PPD/PPA risk due to small cell sizes (e.g., grouping all Asian patients together). Future work should examine differences in positive depression screenings and suicidal ideation across disaggregated Asian patients. Third, the initiative simultaneously transitioned from the PHQ-9 administered verbally by nurses to the EPDS-10 self-administered by patients via iPads. As such, it is not possible to disentangle the impact of changing the screening instrument versus changing the screening modality. Fourth, this study focused on one healthcare institution and the results may not be generalizable to other populations, particularly those that differ from the patient population at Cedars-Sinai. Finally, although the EPDS-10 is reliable and valid in U.S. populations (Moyer et al. 2023a). it was not developed in the U.S. and may have limited cultural suitability for patients of color in particular (Cox 2019). Notably, a revised EPDS-10 for the U.S. was recently developed and should be tested in diverse patient populations (Moyer et al. 2023b).

Despite these limitations, the study has several notable strengths. First, the EMR data do not rely on self-reported mental health outcomes. Second, the EMR data include four years of deliveries at Cedars-Sinai, with a sufficiently large patient population to examine suicidal ideation, which, while rare, is vitally important to maternal health. Third, the EMR data contain several relevant sociodemographic and health characteristics, with sufficient subgroup populations to include these covariates in multivariable models. Finally, because the data are from one hospital, all patients experienced consistent mental health screening processes.

## Conclusion

This study found that a QI initiative replacing the PHQ-9 verbally-administered by nurses with the EPDS-10 self-administered by patients via iPads in the postpartum inpatient setting resulted in higher rates of positive depression screenings, identified suicidal ideation, and social work referrals. These findings are likely due to the use of an instrument specifically designed for postpartum patients as well as the private and independent administration of the screening. Notably, the EPDS-10 captures symptoms of both PPD and PPA, allowing healthcare providers to screen for both PPD and PPA with one instrument. Other healthcare systems may consider implementing similar initiatives to accurately identify patients suffering from PPD/PPA. Mental health screening for PPD and PPA is a vital first step along the pathway to addressing mental health and ultimately preventing maternal morbidity and mortality.
